# Supplementary material for: Content-rich biological network constructed by mining PubMed abstracts
Source: BMC Bioinformatics. 2004 Oct 8;5:147. doi: 10.1186/1471-2105-5-147 (PMC528731; doi:10.1186/1471-2105-5-147)
Supplement: Additional File 2 — The original results of the above study (non-essential files are deleted to keep the file size under the limit set by BMC bioinformatics). [file 1471-2105-5-147-S2.bz2 › chilibotAdditionalFile2/dip05/366ID9045692E6040/html/FGF1_FGFR3.html]

 


 **FGF1** and **FGFR3** 
  
Found 170 abstracts in PubMed, retrieved 05.  
 

 What does Google say? 
 PDF only 
| .edu only 

---

**Interactive relationship** (e.g. stimulation, inhibition, etc)

**Neutral relationship**- Importantly, we also find a number of gene expression changes indicating that FGF  [ **FGF1** ]  promotes many aspects of hypertrophic differentiation, a notion supported by in situ analysis of developing growth plates from mice expressing an activated form of  **FGFR3** .  Ref: 12821644 J Cell Biol< MedlineTA>J Cell Biol,

**Non-interactive relationship** (e.g. studied together, co-existance, homology, etc.)

- RESULTS A large stromal subpopulation expresses fibroblast growth factor receptor 3  [ **FGFR3** ]  FGF  [ **FGF1** ]  R3, indicating a mesenchymal origin of these cells.  Ref: 12865629 PathobiologyPathobiology,
- Activating mutations in FGF  [ **FGF1** ]  receptor 3  **FGFR3**  cause several human dwarfism syndromes by affecting both chondrocyte proliferation and differentiation.  Ref: 12821644 J Cell Biol< MedlineTA>J Cell Biol,
